# Switchable unidirectional waves on mono- and diatomic metamaterials

**DOI:** 10.1038/s41598-022-20972-4

**Published:** 2022-10-07

**Authors:** Jiaruo Yan, Anna Radkovskaya, Laszlo Solymar, Chris Stevens, Ekaterina Shamonina

**Affiliations:** Department of Engineering Science, University of Oxford, Parks Road, Oxford, OX1 3PJ UK

**Keywords:** Engineering, Electrical and electronic engineering

## Abstract

We demonstrate switchable unidirectional propagation of slow waves of coupling within a metamaterial array of strongly coupled elements. We predict theoretically and verify experimentally that the direction of propagation of magnetoinductive waves for any chosen excitation pattern is dictated by the dispersion relations, with forward and backward waves propagating in opposite directions along a chain of meta-atoms. We further prove that the same fundamental phenomenon of direction selectivity due to the forward/backward wave nature is not limited to magnetoinductive waves: we predict analytically and verify numerically the same selective unidirectional signal propagation occurring in nanostructured metamaterial arrays with purely electric coupling. Generalising our method of unidirectional waveguiding to a diatomic magnetoinductive array featuring both forward-wave and backward-wave dispersion branches, switchable unidirectional signal propagation is achieved with distinct frequency bands with opposite directions of signal propagation. Finally, by expanding our technique of selective unidirectional waveguiding to a 2D metasurface, a selective directional control of waves in two dimensions is demonstrated opening up possibilities for directional wireless signal transfer via magnetoinductive surfaces. The observed phenomenon is analogous to polarisation-controlled near-field interference for unidirectional guiding of surface plasmon-polaritons.

## Introduction

Contrary to classical interference of travelling electromagnetic waves observable on a large, wavelength scale, near-field interference can occur in metamaterials on an extremely short scale resulting in novel phenomena of unidirectional waveguiding which may enable fast switching of propagation direction in the near field or radiation direction in the far field. The key to the near-field interference is the vector nature of near-field electromagnetic fields and their ability to interact with slow eigenmodes of a medium. The most prominent examples are based on polarisation-controlled near-field interference of surface plasmon polaritons (SPPs)^1^. The interference requires at least two waves, and a number of possibilities explored recently include use of two closely spaced light sources, or the presence of two different polarisation modes within the same light source, or the presence of a corrugated surface or another kind of structuring of the medium on the nanoscale. Liu et al.^2^ tailored the relative phase and separation between two detuned nanomagnetic antennas that allowed the travelling SPPs to be steered in a desired direction, whereas Rodriguez–Fortuno et al.^3^ achieved unidirectional excitation of guided near-field electromagnetic modes through interference of polarisation components of a circularly polarised dipole. Similarly, methods of engineering of both far- and near-field directionality from dipolar structures with phased combinations of electric and magnetic moments were discussed by Picardi et al.^4^. Nanostructured materials enable strong light-matter interaction resulting in controllable near-field interference of SPPs. Bouillard et al.^5^ demonstrated directional excitation of SPPs via chirped plasmonic gratings. Using arrays of metallic slits patterned into a thin gold film, Lin et al.^6^ presented a method to realise tunable directional coupling of SPP waves while preserving their polarisation, with both bidirectional and unidirectional launching of SPPs. Bisharat et al.^7,8^ demonstrated selective excitation of electromagnetic modes confined and guided along an infinitesimal interface between complementary metasurfaces.

In the present paper we explore the near-field interference of a different kind of slow short waves that can propagate on metamaterials with strong inter-element coupling. We shall consider magnetoinductive (MI) and electroinductive (EI) waves and demonstrate switchable unidirectional propagation using a pair of closely spaced sources yielding the near-field interference of slow waves of coupling. MI waves are short slow waves and they can couple to electromagnetic waves forming polaritons^9–11^ – this makes them analogous to other types of slow eigenmodes of a medium like surface plasma waves or acoustic waves.

The propagation of MI waves relies on the magnetic coupling between elements in the structure. The idea of MI waves was introduced in 2002^12^ in the context of the emerging field of metamaterials as waves that can only propagate within the host magnetic metamaterial by virtue of magnetic coupling between individual resonators. Fundamental properties of MI waves have been studied both theoretically and experimentally in metamaterials comprising magnetically coupled resonant meta-atoms such as split ring resonators (SRRs) or capacitively loaded loops^9,10,13–16^, including also nanostructured metamaterials^17,18^. Applications of MI waves include near-field imaging^19,20^, guiding^21–24^, and sensing^25,26^, wireless data or power transfer^27–29^, transducers^30^, amplifiers for MRI^31^, phase shifters^32^, polarisers^33^, and superdirective antennas^34^.

The dispersion of MI waves is controlled by the coupling coefficient and can feature both forward-wave and backward-wave behaviour^12^. In the presence of retardation, i.e. when the resonators are not small enough, the coupling coefficient can become a complex quantity and can include both magnetic and electric coupling^35^, resulting in more complex dispersion relations^22,36,37^. When meta-atoms are coupled electrically rather than magnetically the established name for slow waves of coupling is electroinductive (EI) waves^38^. Examples are complementary metamaterials in which the metal is replaced by dielectric and vice versa^39^, and nanostructured split-ring media in which the magnetic coupling is largely suppressed due to the so-called kinetic inductance caused by the inertia of electrons^35,40^.

In this paper we shall consider both types of slow waves, MI and EI waves, and derive universal rules for switchable unidirectional propagation for a pair of closely spaced sources yielding near-field interference. We predict theoretically and verify experimentally that the direction of propagation of MI waves for any chosen excitation pattern is dictated by the dispersion relations, with forward and backward waves propagating in opposite directions along a chain of coupled meta-atoms. We further prove that the same fundamental phenomenon of direction selectivity due to the forward/backward wave nature is not limited to MI waves: we predict analytically and verify numerically that the same selective unidirectional signal propagation would be occurring in nanostructured metamaterial arrays with purely electric coupling. Generalising our method of unidirectional waveguiding to a diatomic MI array featuring both forward-wave and backward-wave dispersion branches, switchable unidirectional signal propagation is achieved with distinct frequency bands with opposite directions of signal propagation. Finally, by expanding our technique of selective unidirectional waveguiding to a metasurface, a selective directional control of waves in two dimensions is demonstrated, thus opening up possibilities for directional wireless signal transfer via magnetoinductive surfaces.

The structure of this paper is as follows. In Sect. “Near-field interference of MI waves” we derive analytical conditions for the unidirectional signal guiding based on MI waves interference. In Sect. “Experiments” we verify our model experimentally using chains of capacitively loaded split ring resonators operating in the MHz frequency range. In Sect. “Slow waves of different nature: electroinductive waves in THz arrays” we demonstrate that the same principles of unidirectional guiding apply also to slow waves of a different nature by looking at nanostructured arrays of SRRs in the THz frequency range with predominantly electric coupling. In Sect. “Diatomic case: switchable direction for power flow” we verify the statement that the direction of the signal guiding is dictated by the dispersion characteristics and demonstrate its validity on diatomic chains of SRRs with multiple branches of forward and backward waves. In Sect. “Metasurface example” we expand to two dimensions exploring possibilities of establishing controllable switchable paths for signal propagation on 2D metasurfaces. Conclusions are drawn in Sect. “Conclusions”.

**Figure 1:**
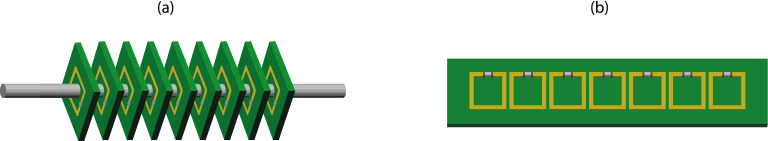
Axial (**a**) and planar (**b**) metamaterial arrays resulting in forward (**a**) and backward (**b**) MI waves .

## Near-field interference of MI waves

Magnetoinductive waveguides consist of resonant elements coupled to each other. A typical example is a line consisting of capacitively loaded resonant loops where the coupling is obviously magnetic. As may be seen in Fig. 1 there are two main configurations, axial and planar.

Assuming that the $n\mathrm{{th}}$ element is coupled to its first, second and third neighbour we may write Kirchhoff’s equation^41^ for $I_n$, the current in the $n\mathrm{{th}}$ element, as

1 $$\begin{aligned} Z_0 I_n +j\omega \sum _{s=1}^{3} M_s (I_{n+s} + I_{n-s}) = 0 \end{aligned}$$

where

2 $$\begin{aligned} Z_0 =j\omega L + \frac{1}{j \omega C} \end{aligned}$$

is the impedance of an element, $\omega$ is the angular frequency, *L* is the self-inductance, *C* is the capacitance, $M_s$ is the mutual inductance between two elements *s* units apart. Note that the elements are assumed to be lossless, because for the phenomena we are interested in, a finite resistance presents only mathematical complications, and has hardly any effect upon the physics. In a finite structure comprising *N* elements, currents in an array can be calculated from the generalised Kirchhoff’s Law,

3 $$\begin{aligned} \mathbf{V} = Z \mathbf{I} \end{aligned}$$

where $\mathbf{V}$ and $\mathbf{I}$ are *N*-dimensional vectors standing for the applied voltages and for the resulting currents, and *Z* is the impedance matrix the main diagonal elements being all equal to the self-impedance $Z_0$ and the off-diagonal entries being the mutual impedances between elements of the array.

An alternative approach is to invoke the wave concept. A solution of the difference Eq. (1) may be obtained as

4 $$\begin{aligned} I_n = I_0 \text {e}^{-jknd} \end{aligned}$$

where *d* is the separation between the elements, $I_0$ is a constant and *k* is the wave number. The wave number is real within the passband of MI waves due to our assumption of negligible losses. The solution is valid when the frequency, $\omega$, and the wave number, *k*, are related to each other by the dispersion equation

5 $$\begin{aligned} \frac{\omega _0^2}{\omega ^2} -1 = \sum _{s=1}^{3} \kappa _s \cos (skd) \end{aligned}$$

where $\omega _0=1/\sqrt{LC}=2\pi f_0$ and $f_0$ is the resonant frequency of each unit cell and $\kappa _s = 2M_s/L$ are the coupling constants. The dispersion equation can has two notable solutions, a forward wave in the axial configuration when $M_s > 0$ and a backward wave in the planar configuration when $M_s < 0$. If we apply a voltage, to any of the elements, then magnetoinductive waves will propagate in both directions (with the obvious exception when the excited element is the first or the last one in the line). Our main interest is to control the relative amount of power propagating in the two directions. For that we need to excite at least two elements and should be free to choose their relative phases.

Our model is a linear array of *N* identical elements, numbered from left to right, elements *p* and $p+1$ near the centre of the array are excited with voltage sources of the same amplitude but with a phase difference of $\psi$, i.e. $V_p=\exp (j\psi )$ and $V_{p+1}=1$. If we consider the currents in elements $p - 1$ and $p + 2$ that are on opposite sides of the excited elements, the expressions for the currents $I_{p-1}$, $I_{p+2}$, can be found based on superposition of the waves launched from elements *p* and $p + 1$ as

6 $$\begin{aligned} |I_{p-1} |= & {} |\text {e}^{j\psi } + \text {e}^{-jkd}| \end{aligned}$$

7 $$\begin{aligned} |I_{p+2}|= & {} |\text {e}^{j\psi } + \text {e}^{jkd} | . \end{aligned}$$

**Figure 2:**
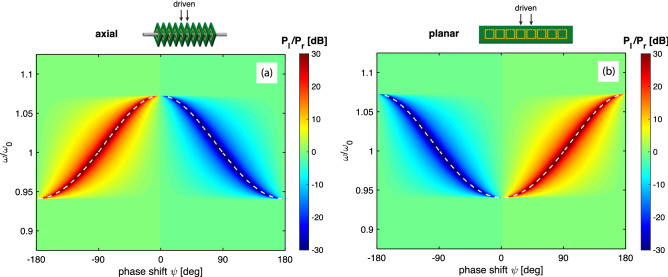
Power flow ratio $P_\mathrm{l}/P_\mathrm{r}$ for an axial array with $\kappa =0.13$ (**a**) and a planar array with $\kappa =-0.13$ (**b**). $N=27$. Lossless case. Analytical model. Excitation in the central elements 14 and 15, $V_{14}=\exp (j\psi )$ and $V_{15}=1$. Elements 1 and 27 are matched to eliminate reflections from the ends. White dashed line corresponds to the analytical conditions of unidirectional power flow from Eqs. (10 and 11).

There will be reflections from both ends of the array that may be considerable in the absence of losses. The remedy is to terminate both ends with impedances which may offer perfect matching. The matching impedance is given by^41^

8 $$\begin{aligned} Z_T = j\omega \sum _{s=1}^{3} M_s \text {e}^{-jskd} \end{aligned}$$

which becomes purely resistive at the resonant frequency $kd=\pi /2$ within the nearest-neighbour approximation. In order to have a better idea of the effect of the chosen phase on the ratio of powers we shall have a more detailed look at the expression

9 $$\begin{aligned} \frac{P_\mathrm{{l}}}{P_\mathrm{{r}}}=\frac{P_{p-1}}{P_{p+2}} = \frac{|I_{p-1}|^2}{|I_{p+2}|^2} = \frac{\cos ^2[(\psi +kd)/2]}{\cos ^2[(\psi -kd)/2]} \end{aligned}$$

where $P_\mathrm{{l}}$ and $P_\mathrm{{r}}$ stand for the power propagating to the left and to the right respectively. A complete suppression of the power flow to the left corresponds, according to Eq. (9) to the condition

10 $$\begin{aligned} \psi =-kd \pm \pi \end{aligned}$$

whereas a complete suppression of the power flow to the right corresponds to the condition

11 $$\begin{aligned} \psi =kd \pm \pi \end{aligned}$$

where *kd* is dictated by the dispersion Eq. (5). In particular, if we were to fix the value of the phase shift $\psi$ between the voltage sources, the preferential power flow will be opposite in an axial array and in a planar array. In other words, a forward-wave structure and a backward-wave structure will guide the power in opposite directions. This is detailed in Fig. 2, in which the variation of $P_\mathrm{{l}}/P_\mathrm{{r}}$ is shown as a contour plot on the $(\psi ,\omega )$ parametric plane using typical parameters of an axial MI array with a positive coupling coefficient $\kappa = 0.13$ (a) and a planar MI array with a negative coupling coefficient $\kappa =-0.13$ (b) and assuming no reflections from the ends of the array. The red colour corresponds to the power flow to the left, and the blue colour corresponds to the power flow to the right. For the phase shift in the positive range of $0< \psi < \pi$, the axial array [Fig. 2a] enables the power flow mostly to the right and the planar array [Fig. 2b] enables the power flow mostly to the left. Changing the phase shift to the negative range of $-\pi< \psi < 0$ the directions of the power flow switch. As will be shown below this is a fundamental feature which links the type of the dispersion (forward/backward) and the direction of the power flow suppression. White dashed lines show the conditions of the complete suppression of the power flow as dictated by Eqs. (10 and 11) and they can be seen to accurately predict the phase shift-frequency relationship for maximum unidirectional power flow.

In the lossy case, the self-impedance of the resonators, $Z_0=R+j\omega L + 1/(j\omega C)$ includes the resistance *R*, and the dispersion Eq. (5) modifies to

12 $$\begin{aligned} \frac{\omega _0^2}{\omega ^2} -1 +j\frac{\omega _0}{\omega Q}= \sum _{s=1}^{3} \kappa _s \cos (skd) \end{aligned}$$

where $Q=\omega _0L/R$ is the quality factor of each resonator. In the lossy case the wave number $k=\beta -j \alpha$ is a complex number, with $\beta$ being the propagation constant and $\alpha$ being the attenuation constant, however Eq. (9) is still valid and Eqs. (10 and 11) will only mildly be affected by the attenuation within the passband of MI waves.

## Experiments

**Figure 3:**
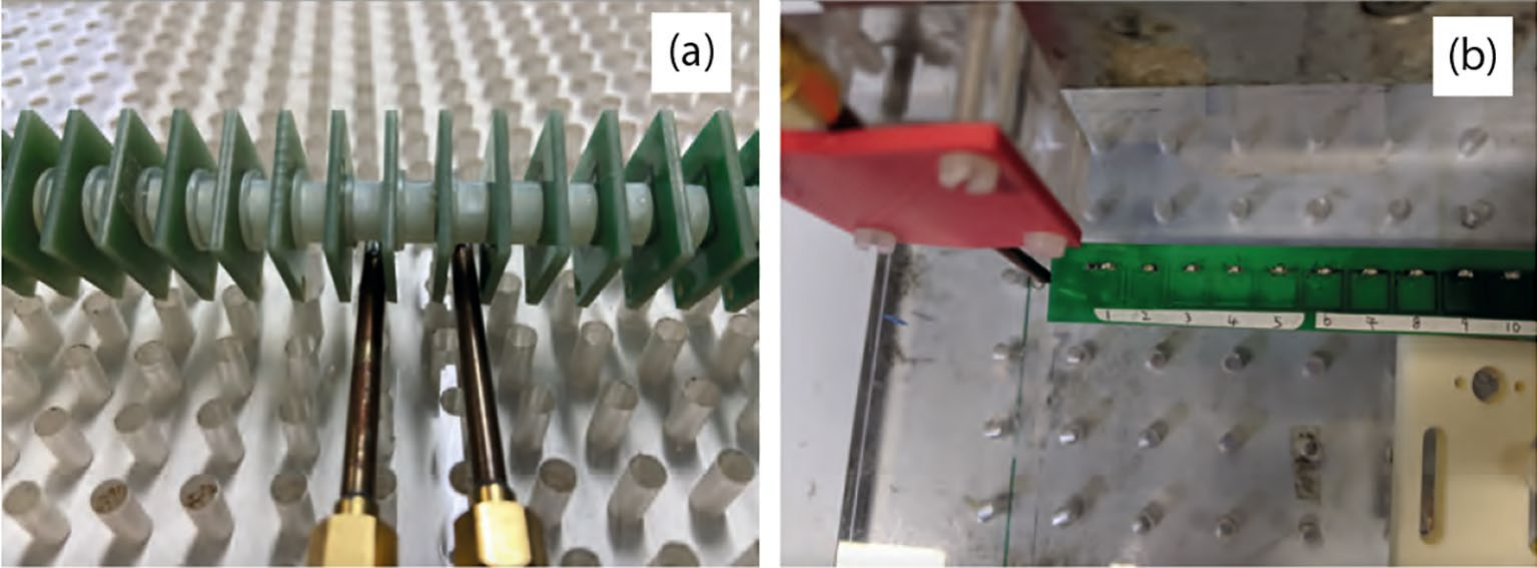
Photographs of the axial (**a**) and planar (**b**) structures used in the experiments. Small loop antennas under the central elements 14 and 15 are used to excite the array, while the receiving antenna is moved across the structure.

To verify our model, we use a planar and an axial array of 27 strongly coupled square-shape meta-atoms. Each meta-atom is a PCB-based square resonator of 10 mm side length, 1 mm track width, made of 1 oz copper, and has a 100 pF capacitor soldered into a 1mm gap to tune the resonant frequency to $f_0 = 116.6$ MHz (axial array) and $f_0=114.1$ MHz (planar array). The substrate is 1.6 mm FR4 TG130. The substrate size for the unit cells in the axial array is 19 mm $\times$ 19 mm, and the measured quality factor for a single element is $Q = 80$. The photographs of the axial and planar configurations used in the experiments are shown in Figs. 3a and b, respectively.

The strength of the mutual coupling between meta-atoms depends on the separation between them. The lattice periods for the two configurations were designed to provide the nearest neighbour coupling coefficients that are equal in magnitude in both arrays, $\kappa _1$, for the axial and planar arrays. The lattice period *d* for the axial array is 6.6 mm, resulting in positive coupling coefficients, $\kappa _1=0.13$, $\kappa _2 = 0.034$ and $\kappa _3 = 0.017$. For the planar array, the lattice period is *d* = 10.5 mm resulting in negative coupling coefficients $\kappa _1=-0.13$, $\kappa _2 = - 0.0062$ and $\kappa _3 = -0.0004$.

We performed two sets of measurements, first, a standard dispersion relationship analysis and, second, the near-field interference measurements. To obtain the dispersion curve, we use an established technique in which the structure is excited at element 1 by a loop antenna of 4 mm diameter connected to a vector network analyser (VNA). The currents in three adjacent elements $(n-1)$, *n* and $(n+1)$ are then measured yielding the complex wave number $k=\beta -j\alpha$ as follows:

13 $$\begin{aligned} \cos (\beta d - j \alpha d)= \frac{I_{n-1}+I_{n+1}}{2I_n}. \end{aligned}$$

**Figure 4:**
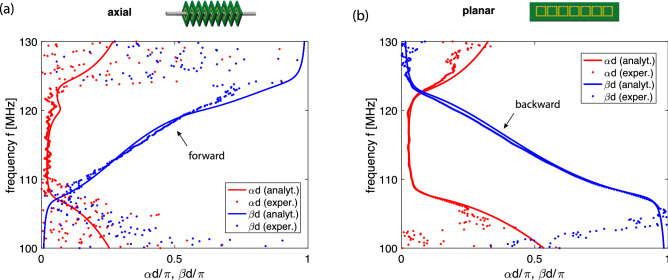
MI dispersion relations for the axial (**a**) and the planar (**b**) array. Red: attenuation constant $\alpha$ vs. $\omega$, blue: propagation constant $\beta$ vs. $\omega$. Analytical model (solid lines) and experimental data (dots).

The normalised values of the currents are obtained by scanning the array with a receiving loop antenna of 4 mm diameter and measuring the scattering parameter $S_{21}$ proportional to the currents in the array. The experimentally found dispersion is shown in Fig. 4 (dots). Within the passband the experimental data can be seen to be in agreement with the theoretical model (solid lines) obtained from Eq. (12) using the above parameters for the resonant frequency, the quality factor and the coupling constants. While we are primarily interested in the passband, we note that in the stopband the experimental data show some outliers. The reason is that the signals in the stopband are too weak to enable reliable dispersion extraction.

The dispersion curves for both arrays confirm clearly the forward-wave nature for the axial array (a) and the backward-wave nature for the planar array (b) whereas the passband in both arrays are approximately equal due to the same magnitude of the dominant coupling coefficient in both arrays.

**Figure 5:**
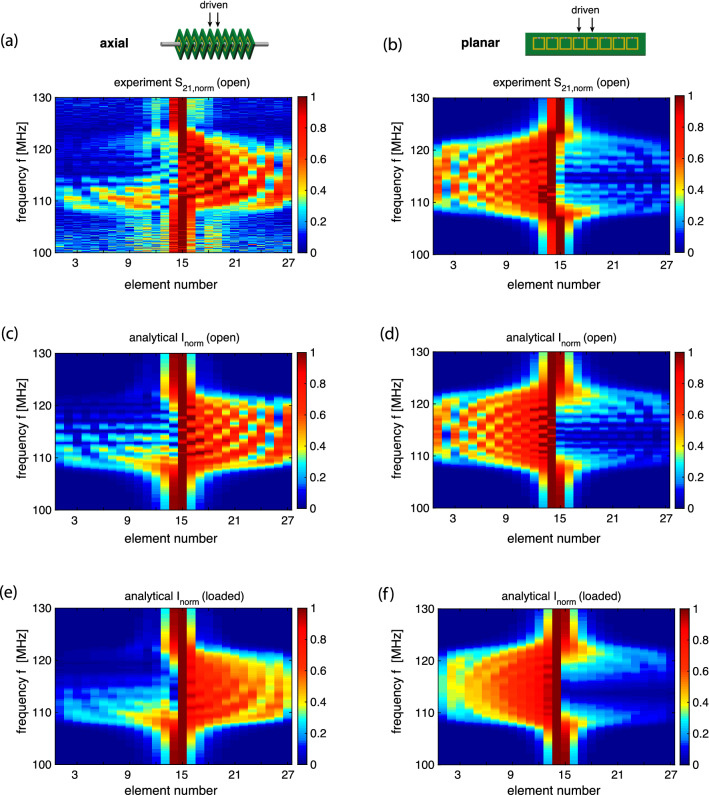
The normalised current distribution in the 27 elements of the MI waveguides over frequency, for an axial array (left column) and for a planar array (right column) shown schematically in the insets, with elements 14 and 15 excited as indicated by arrows. (**a**), (**b**): spectra for the magnitude of $S_{21}$ (experiment). (**c**)–(**f**): spectra for the current magnitude (analytical model). (**a**)–(**d**): arrays are open (not loaded). (**e**), (**f**): both ends of the array are loaded to suppress reflections.

Next we performed the near-field interference experiment exciting two central elements (elements 14 and 15) with a constant phase shift $\psi =\pi /2$ by using two loop antennas (of 4 mm diameter) connected to a VNA via a hybrid 2 power splitter (Mini-Circuits, ZMSCQ-2-120+). Using the same procedure as before, a receiving antenna performed a scan of the array and currents in all elements of the array were obtained and compared to the theoretical calculations. The results for the axial and the planar array are summarised in Fig. 5 (left column: axial array, right column: planar array). The experimentally found current distributions for both arrays are shown in Fig. 5a and b as contour plots with the horizontal axis being the element number (from 1 to 27) and the vertical axis being the frequency. The corresponding theoretical results from Eq. (3) shown in Fig. 5c and d can be seen to reproduce all salient features of the experimental data. These results confirm clearly the main prediction of our model: near-field interference results in a preferential signal propagation to the right in the axial array and to the left in the planar array. The interference pattern caused by reflections from the ends of the structure can be suppressed by using at the resonant frequency approximate matched loads: $Z_\mathrm{T} = 1 \Omega$ for the axial array, and $Z_\mathrm{T} = 0.9 \Omega$ for the planar array. With the reflections significantly suppressed, the current distributions in Figs. 5e and f reveal unilateral signal propagation around the resonant frequency, in accordance with the dispersion relations and selection rules of Eqs. (10 and 11).

## Slow waves of different nature: electroinductive waves in THz arrays

**Figure 6:**
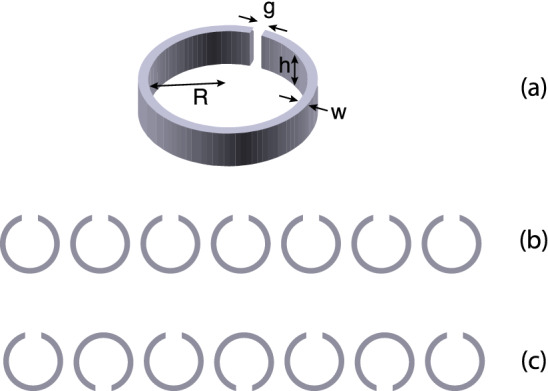
Schematic for (**a**) a single SRR element with inner radius *R*, width *w*, height *h*, and gap *g*, (**b**) a linear ‘uu’ array, which has positive nearest-neighbour coupling coefficient and (**c**) a ‘un’ array which has negative nearest-neighbour coupling coefficient.

Next we shall investigate whether the conclusions reached so far are also valid in another much higher frequency band and for a different coupling mechanism between the elements. We can test this by venturing into the THz region, which enables a different type of slow waves of coupling, electroinductive waves^35^. In a nanostructured array of split ring resonators, if the frequency is sufficiently high and the structure sufficiently small, the magnetic coupling is largely suppressed due to electrons inertia and the remaining coupling mechanism is the electric coupling^35^. The dispersion relationship for EI waves can be written as

14 $$\begin{aligned} \frac{\omega ^2}{\omega _0^2} -1 +j\frac{\omega }{\omega _0 Q}= \sum _{s=1}^{3} \kappa _{\mathrm{E},s} \cos (skd) \end{aligned}$$

where $\kappa _{\mathrm{E},s}=2C/K_s$ is the electric coupling coefficient and $K_s$ is the mutual capacitance between meta-atoms *s* lattice periods apart. Similarly to their MI counterpart, EI waves can also yield forward and backward waves depending on the sign of the coupling coefficient. We shall consider two types of geometries as shown in Fig. 6, but instead of experiments (THz equipment is not easily available) we shall use numerical simulations (CST Microwaves Studio) to confirm the analytical results.

The unit cell we use is a silver SRR as shown in Fig. 6a with inner radius $R = 80$ nm, width $w = 8$ nm, height $h = 40$ nm, and the gap width $g =12$ nm. We use the Drude model with plasma frequency, $\omega _\mathrm{p} = 1.35 \cdot 10^{16}$ rad/s, and collision frequency, $\gamma = 33$ THz to evaluate the permittivity of silver^35^. These parameters yield the resonant frequency of a single element, $f_0 = 93.5$ THz, and the quality factor, $Q = 25$.

We use two different arrangements of the split rings, shown in Figs. 6b and c, in order to find both forward-wave and backward-wave behaviour. When all SRRs in the array have gaps facing upwards [Fig. 6b] the nearest coupling constant is almost real positive (with a small phase due to retardation). This configuration will be called the ‘uu’ configuration (as each of the elements resembles the letter ‘u’). By flipping every second element so that their gaps are now facing downwards [Fig. 6c] the nearest coupling constant becomes almost real negative, with a small phase due to the retardation. We call this a ‘un’ configuration (as now the elements resemble an alternating sequence of the letters ‘u’ and ‘n’). We chose for both arrays the same lattice period $d = 192$ nm and the same number of elements, $N=27$.

**Figure 7:**
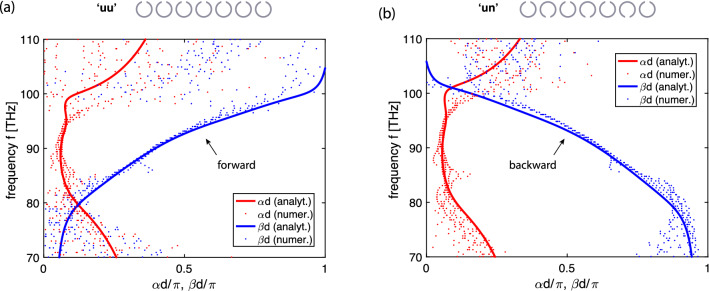
EI dispersion relations for the ‘uu’ (**a**) and the ‘un’ (**b**) array. Red: attenuation constant $\alpha$ vs. $\omega$, blue: propagation constant $\beta$ vs. $\omega$. Analytical model (solid lines) and numerical data (dots).

**Figure 8:**
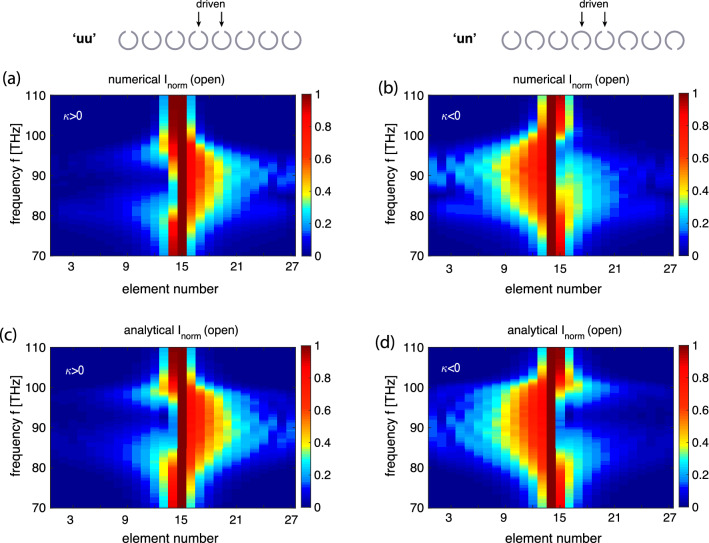
The normalised current distribution in the 27 elements of the EI waveguides over frequency, for the ‘uu’ array (left column) and for the ‘un’ array (right column) shown schematically in the insets, with elements 14 and 15 excited as indicated by arrows. (**a**), (**b**): numerical simulation. (**c**), (**d**): analytical model .

The resulting dispersion relations are shown in Fig. 7 confirming the expected forward-wave character for the ‘uu’ array (a) and the backward-wave character for the ‘un’ array (b). The analytical curves (solid lines) are calculated from Eq. (14) using $f_0=91.5$ THz and $\kappa _{\mathrm{E},1,2,3}=[-0.2\text {e}^{j\varphi _1},\ -0.025\text {e}^{j\varphi _2},\ -0.015 \text {e}^{j\varphi _3}]$ (‘uu’ configuration) and using $f_0=92$ THz and $\kappa _{\mathrm{E},1,2,3}=[0.22\text {e}^{j\varphi _1},\ -0.025\text {e}^{j\varphi _2},\ 0.016 \text {e}^{j\varphi _3}]$ (‘un’ configuration), where $\varphi _s =s \cdot 0.09$ rad is the coupling coefficient phase due to retardation. The numerical dispersion curves are obtained following the same extraction procedure as the one used for post-processing experimental data for neighbouring currents, i.e. by using Eq. (7), and with the current values evaluated from signals in magnetic probes placed near the centres of the elements. As the magnitudes of the dominant coupling constants are similar in both arrays, it can be seen from Fig. 7 that their passband occupy the same frequency band from 80 to 100 THz. To perform the near-field interference test for EI waves, we follow the same recipe as before. Two central elements (elements 14 and 15) are driven with a phase shift of $\psi =\pi /2$ applying $V_{14}=\exp (j\pi /2)$ and $V_{15}=1$ V and the frequency spectra for all currents along the array are recorded. The results are summarised in Fig. 8, with the left column showing the results for the forward-wave ‘uu’ array, and the right column for the backward-wave ‘un’ array. Numerically obtained current distribution for both arrays are shown in Fig. 8a and b as contour plots with the horizontal axis being the element number (from 1 to 27) and the vertical axis being the frequency. The corresponding analytical results shown in Fig. 8c and d can be seen to reproduce all salient features of the simulation data. In comparison to the MI wave case of Fig. 5, there are no visible ripples due to reflections from the ends of the arrays as the THz structures are rather lossy and the signal decays too much along the line to yield any noticeable reflection. Importantly, the main prediction of our model is validated: also for the electroinductive waves, the near-field interference results in a preferential signal propagation to the right in the forward-wave ‘uu’ array and to the left in the backward-wave ‘un’ array.

## Diatomic case: switchable direction for power flow

**Figure 9:**
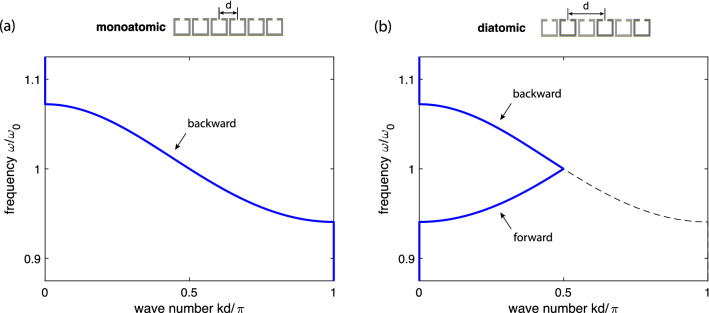
An example of a lossless MI dispersion of a monoatomic metamaterial structure with $\kappa =-0.13$ (**a**) and its degenerate diatomic counterpart showing doubling the lattice constant *d* and the resulting folding of the dispersion forming the double-band frequency structure with a forward and a backward wave within the 1st Brillouin zone.

Having verified the main feature of the near-field interference of slow MI and EI waves, namely that the direction of signal propagation is dictated by whether the wave is a forward or a backward wave, we shall now use diatomic MI arrays to implement a switchable device. The key thought is that diatomic chains, with two resonators constituting a unit cell, are proven to exhibit two passbands of slow waves^23,42^. We shall look at diatomic arrays featuring two bands of the opposite wave type, one band with a forward-wave dispersion and another one with a backward-wave dispersion. Switching between two bands should enable switching of the direction of the power flow.

There are two obvious ways of implementing the diatomic structure, (i) to use two types of meta-atoms, say, having different resonant frequencies, $f_{01}$ and $f_{02}$, and thus different self-impedances, $Z_{01}$ and $Z_{02}$ and (ii) to alternate the distance between the elements from $d_1$ to $d_2$ thus having two alternating mutual inductances $M_1$ and $M_2$^41^. The dispersion equation derived for either of the cases

15 $$\begin{aligned} \cos \left[ \frac{k(d_1+d_2)}{2}\right] = \frac{1}{2}\frac{ \sqrt{\displaystyle -\frac{Z_{01}Z_{02}}{\omega ^2} -(M_1-M_2)^2 }}{\sqrt{M_1M_2}} \end{aligned}$$

shows the existence of two passbands. Interestingly, using both the axial and the planar array results in the lower band being a forward wave branch and the upper band being a backward wave branch. It is important to note that the degenerate case (i. e. the case with $Z_{01}=Z_{02}=Z_0$, $d_1=d_2=d$ and $M_1=M_2=M$) is equivalent to a monoatomic array, so both descriptions of a monoatomic array are compatible, taking into account that the lattice period in the diatomic array is twice that of the monoatomic array, and using the usual rules of folding for Brillouin zones for waves in periodic structures^43^. This is illustrated in Fig. 9 using a planar array as an example. Figure 9a shows the familiar backward-wave dispersion of a monoatomic planar MI array, with the lattice constant *d*, with the Brillouin zone range of *kd* from 0 to $\pi$. Figure 9b shows the degenerate diatomic dispersion for the same array, with the lattice constant *d* now doubled and the dispersion seen to have folded into the $1^\mathrm{st}$ Brillouin zone forming a forward-wave and a backward-wave branch.

**Figure 10:**
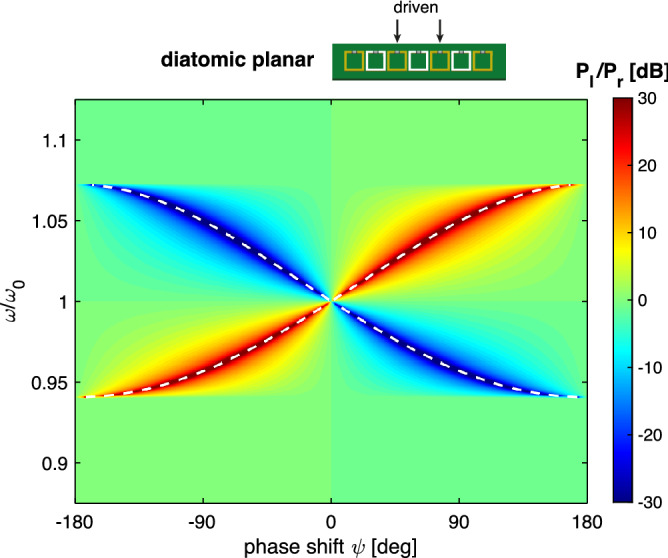
Power flow ratio $P_\mathrm{l}/P_\mathrm{r}$ for a diatomic excitation ($V_{13}=\exp (j\psi )$ and $V_{15}=1$) of a planar array with $\kappa =-0.13$ (shown by arrows in the inset). Length of the array $N=27$. Lossless case. White dashed line corresponds to the analytical conditions of unidirectional power flow from Eqs. (10 and 11).

**Figure 11:**
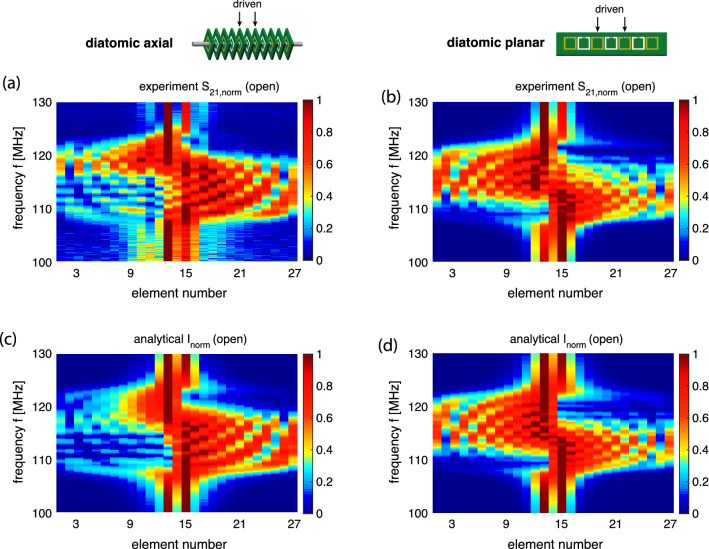
Switchable unidirectional power flow. The normalised current distribution in the 27 elements of the MI waveguides over frequency for an axial array (left column) and for a planar array (right column). (**a**),(**b**): spectra for the magnitude of $S_{21}$ (experiment). (**c**),(**d**): spectra for the current magnitude (analytical model). Array is open (not loaded). Diatomic excitation of elements 13 and 15 [$V_{13}=\exp (j\pi /2)$ and $V_{15}=1$] is shown schematically by arrows in the insets.

This leads us to a simple possibility for implementing a switchable power flow. Treating monoatomic arrays as degenerate diatomic arrays, we shall implement near-field interference exciting not two neighbouring meta-atoms, but two neighbouring unit cells instead. In this case the analysis of Eq. (11) is still valid. In Fig. 10 we show the variation of $P_\mathrm{l}/P_\mathrm{r}$ as a contour plot on the $(\psi ,\omega )$ parametric plane for the the planar array with $N=27$, excitation on elements 13 and 15 with a phase shift of $\psi$ and using the same coupling coefficient as in Fig. 2 (b), $\kappa =-0.13$ and assuming again that reflections from the ends are eliminated by using the matched loads. Figure 10 verifies that by exciting non-adjacent elements the near-field interference would indeed enable the switching of the power flow directions.

For the phase shift in the positive range of $0<\psi <\pi$, if the frequency is below the resonant frequency, the power would flow mostly to the right (corresponding to the forward-wave type), but switching to a frequency above the resonant frequency would enable the power to flow mostly to the left (corresponding to the backward-wave type). Changing the phase shift to the negative range of $-\pi<\psi <0$ swaps the directions of the power flow. White dashed lines show the conditions of the complete suppression of the power flow as dictated by Eqs. (10 and 11) and they can be seen to accurately predict the phase shift-frequency relationship for the unidirectional power flow.

We have carried out the experiment confirming the switchable power flow – the setup is exactly the same as before, with the only difference being that elements 13 and 15 are now being excited ($V_{13}=\exp (j\pi /2)$, $V_{15}=1$). The results are summarised in Fig. 11 both for the axial and for the planar array. The experimentally found current distributions for both arrays are shown in Fig. 11a and b and the corresponding theoretical results from Eq. (3) are shown in Fig. 11c and d and can be seen to match well the experimental data.

As can be seen, by exciting non-adjacent elements 13 and 15 with the right phase difference, our array can be regarded as a diatomic one which can support both a forward wave and a backward wave. In the lower-frequency forward-wave band the power travels to the right end and in the higher-frequency backward-wave band the power travels towards the opposite, left end of the structure. The switch occurs when the frequency changes. Potentially, this phenomenon can have application as a channel dropping filter.

## Metasurface example

**Figure 12:**
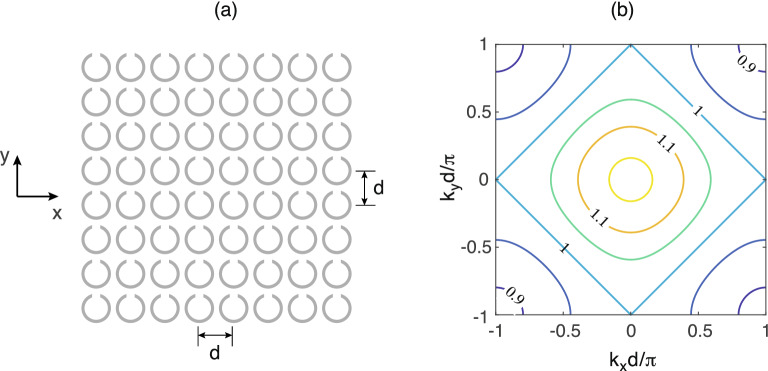
A schematic of a planar isotropic metasurface (**a**) and its lossless MI dispersion (iso-frequencies of $\omega /\omega _0$ vs $k_x$ and $k_y$) for $\kappa _x=\kappa _y=-0.13$ (**b**).

**Figure 13:**
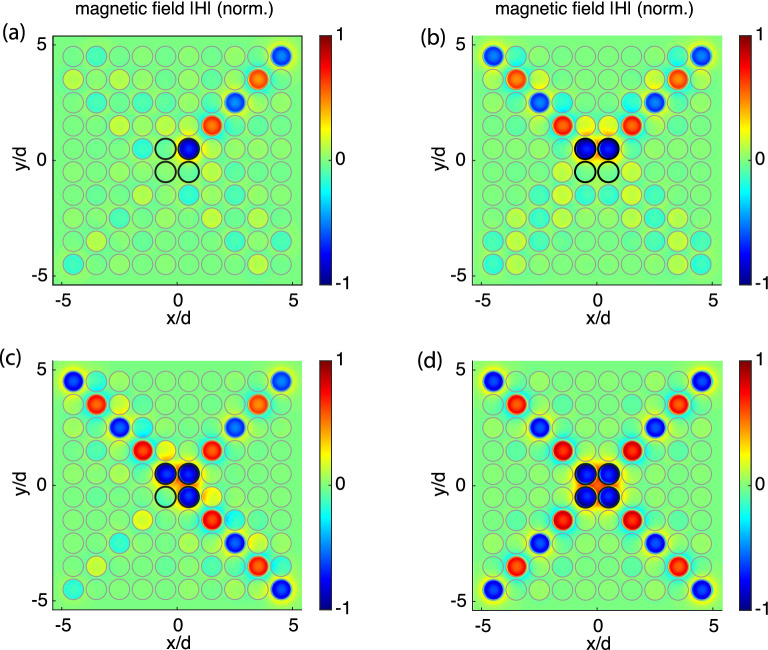
Switchable unidirectional power flow in a metasurface at $\omega =\omega _0$. Magnetic field (normalised) above a 10 $\times$ 10 planar metasurface, with four central elements driven (marked as black circles) to yield a single beam (**a**), two beams (**b**), three beams (**c**) and four beams (**d**). Analytical model for $\kappa _x=\kappa _y=-0.13$, $Q=80$ and using circular-shaped elements, shown with grey lines.

So far we have considered 1D arrays. Can we employ the near-field interference selectivity for controlling signal propagation in 2D metasurfaces? If we expand to two dimensions, the physics of MI waves does not change, the MI wave propagate in the same way as in 1D arrays, by means of coupling between the elements. Taking an isotropic planar array with a square lattice of $d \times d$ shown schematically in Fig. 12 (a) as an example, each element is coupled to its neighbours in both the horizontal (*x*) and the vertical (*y*) direction. The coupling constants in both directions are negative and equal, $\kappa _x=\kappa _y$. The dispersion of the MI waves for such a metasurface can be written in the form^41^

16 $$\begin{aligned} \frac{\omega _0^2}{\omega ^2} -1 +j\frac{\omega _0}{\omega Q}= \kappa _x \cos (k_xd) + \kappa _y \cos (k_yd) \end{aligned}$$

with $k_x$ and $k_y$ being the components of the wave vector $\mathbf{k}$. The 2D dispersion equation is illustrated in Fig. 12b using $\kappa _x=\kappa _y=-0.13$ as an example. The isofrequency dispersion curves show that at every frequency multiple waves can propagate with the relationship between $k_x$ and $k_y$ dictated by Eq. (16). We shall take as an example the resonant frequency $\omega =\omega _0$ in which the isofrequency in Fig. 12b can be seen to form straight lines with a simple relationship between the wave vector components $|k_xd \pm k_yd|=\pi$. A symmetric excitation in the centre of such an array results in signal travelling towards all four corners of the structure forming the ‘diagonal beaming’ as reported previously^41^. Figure 13 illustrates that, by exciting just a small central section of $2 \times 2$ resonators in a symmetric $10 \times 10$ array, we can successfully suppress any of the four diagonal beams thus achieving unidirectional signal propagation in the desired direction. In Fig. 13 we show the normal (*z*) component of the magnetic field distribution above the metasurface, which reflects the current distribution across the array for the four chosen excitation patterns. To impose single-ray beaming of Fig. 13a, the four central elements were excited with the voltage pattern

17 $$\begin{aligned} V=\left( \begin{array}{cc} q &{}1 \\ 1&{} q \end{array}\right) \end{aligned}$$

where $q=3.45\exp (j\pi /2)$. Multiple beams in Fig. 13b–d are obtained by superposition of the corresponding patterns of Eq. (17) rotated by 90 degrees. We point out that our goal in this section is to solely demonstrate the principal possibility of signal control by near-field interference in metasurfaces and that no attempt was made to use matching terminal loading to any of the elements. Not shown here but also successfully tested is a similar beaming suppression in arrays with odd number of elements, albeit with a more extended ‘excitation island’ of up to $3 \times 3$ elements in the centre of the array, i. e. the central element and its neighbours. We conjecture that by moving to a different isofrequency of the dispersion equation of Fig. 12b and using appropriate load impedances would enabling beaming in any desired direction within a metasurface thus enabling an efficient way of wireless signal and power transfer, but this would be a subject of a future investigation.

## Conclusions

Unidirectional wave guidance resulting from near-field interference is well established in the literature for a variety of scenarios for slow waves of surface-plasmon polaritons. We show that an analogous effect can also be realised with a different type of slow waves – with slow waves of coupling in metamaterials. Using as examples magnetoinductive waves (in the MHz frequency range) and electroinductive waves (in the THz frequency range) we prove that switchable unidirectional signal propagation is dictated by the dispersion relations of the slow waves, with cancellation direction determined by whether the waves are forward or backward. Selectivity rules based on near-field interference have been verified experimentally for MI waves and numerically for EI waves. Extending the analysis to diatomic arrays we demonstrate the possibility of simultaneous bands of waves of both types (forward/backward) which enable frequency-controlled switching of unidirectional signal propagation. We successfully apply our technique of selective unidirectional waveguiding to 2D metasurfaces, demonstrating selective directional control of waves in two dimensions enabling an efficient mechanism for directional wireless signal transfer or power transfer. The observed phenomenon is analogous to near-field interference for unidirectional guiding of surface plasmon-polaritons and it is safe to assume that any other type of slow waves could be manipulated enabling controlled and switchable near-field signal guiding on the subwavelength scale.

## Data Availability

The data that support the findings of this study are available from the authors upon reasonable request.
